# Reduction of neck pain severity in patients with medication-overuse headache

**DOI:** 10.1186/s10194-024-01876-2

**Published:** 2024-11-04

**Authors:** Yooha Hong, Hong-Kyun Park, Mi-Kyoung Kang, Sun-Young Oh, Jin-Ju Kang, Heui-Soo Moon, Tae-Jin Song, Mi Ji Lee, Min Kyung Chu, Soo-Jin Cho

**Affiliations:** 1 Department of Neurology, Dongtan Sacred Heart Hospital, Hallym University College of Medicine, 7, Keunjaebong-gil, Hwaseong-si, Gyeonggi-do 18450 Korea; 2 Department of Neurology, Inje University Ilsan Paik Hospital, Inje University College of Medicine, Goyang, Korea; 3 Department of Neurology, Jeonbuk National University Hospital, Jeonbuk National University School of Medicine, Jeonju, Korea; 4 Department of Neurology, Kangbuk Samsung Hospital, Sungkyunkwan University School of Medicine, Seoul, Korea; 5 Department of Neurology, Seoul Hospital, Ewha Womans University College of Medicine, Seoul, Korea; 6 Department of Neurology, Seoul National University Hospital, Seoul National University College of Medicine, Seoul, Korea; 7 Department of Neurology, Severance Hospital, Yonsei University College of Medicine, Seoul, 03722 Korea

**Keywords:** Neck pain, Medication overuse headache, Migraine, Disability

## Abstract

**Background:**

Neck pain and primary headache disorders are highly prevalent in populations and clinical cohorts. Medication-overuse headache (MOH) is a treatable secondary headache, mainly developing in migraine sufferers, that accounts for the majority of patients presenting to headache clinics. Nevertheless, the association between neck pain and MOH has not been reported. This study evaluated the prevalence and clinical course of neck pain in patients with MOH before and after MOH treatment.

**Methods:**

We analyzed 635 MOH patients enrolled in a nationwide, prospective, multicenter MOH registry. Demographics and clinical data were collected at baseline and 3 months to evaluate changes in the status and severity of neck pain and headache. Severity of neck pain was graded into 4 groups, and severe neck pain was defined as grade 3 or 4.

**Results:**

Among 635 patients with MOH, 366 (57.6%) reported neck pain at baseline. MOH patients with neck pain had an earlier onset of their primary headache disorder (23.4 ± 12.7 vs. 26.2 ± 13.3 years, *p* = 0.007). Although monthly headache days were comparable between the patients with neck pain and those without neck pain, the neck pain group had higher levels of anxiety (7.4 ± 5.8 vs. 6.4 ± 5.4, *p* = 0.017), more severe cutaneous allodynia (2.4 ± 3.3 vs. 1.8 ± 3.0, *p* = 0.038), and poorer quality of life (171.7 ± 70.4 vs. 184.0 ± 68.9, *p* = 0.029). At 3 months, 456 (71.8%) were followed-up, and 257 (56.4%) were recovered from MOH. Compared to the baseline, the proportion of severe neck pain (40.4% vs. 19.4%, *p* < 0.001) was decreased. The proportion of severe neck pain was much lower in patients with recovery from MOH compared to those without (4.7% vs. 15.1%, *p* < 0.001).

**Conclusions:**

Neck pain in MOH patients was associated with earlier onset of headache, higher levels of anxiety and allodynia, and poorer quality of life. Improvement in neck pain improvement was linked to recovery from MOH. These findings suggest the potential importance of integrating and management of neck pain into clinical practice for MOH.

## Background

Neck pain is a common symptom frequently experienced by individuals with various types of headaches. Previous studies have shown that the high prevalence of neck pain in migraine patients ranges from 51.9 to 77% in both general population and clinical settings [1–3]. Previous studies highlighted the close association between migraine, tension-type headache, and neck pain, suggesting common underlying mechanisms, triggering factors or a common concurrent symptom [4–9]. The Global Burden of Disease Study ranked neck pain as the 19th leading cause of disability-adjusted life years among young adults [10]. When combined with migraine, neck pain can substantially increase the disability and impact on quality of life for affected individuals [3]. However, it remains unclear whether neck pain is a manifestation related to headache disorders, a co-existing musculoskeletal problem, a result of chronification due to repetitive attacks, or a combination of these factors [4, 11–15].

Medication-overuse headache is a secondary headache disorder caused by regular frequent consumption of acute symptomatic medications in patients with primary headache disorders [16, 17]. Although the association between primary headache disorders and neck pain is well recognized, the relationship between neck pain and medication overuse headache (MOH), a highly disabling treatable secondary headache disorder, has received relatively less attention in clinical research [18]. MOH represents a significant proportion of the global disease burden of headache disorder [19] and is characterized by the overuse of headache medications in patients with migraine, tension-type headache or cluster headaches, leading to worsening headache symptoms, even in pediatric ages [20–22]. Patients with MOH are commonly encountered up to 70% in headache clinics [3, 23] and often present as challenging cases to manage, yet the impact and clinical course of neck pain in MOH remains relatively unexplored and poorly understood.

By examining the prevalence and potential implications of neck pain in MOH patients, this research seeks to elucidate the intricate interplay between headache disorders and associated symptoms. The aim of this study was to assess the frequency and severity of neck pain following treatment for MOH by prospectively registering patients in the MOH registry and determining if there is any potential for improvement. Since there has been no study which examined the neck pain in patients with MOH, these insights could inform more effective management strategies that address both the primary headache disorder and associated neck pain, ultimately improving patient outcomes and quality of life.

## Methods

### Study population

The Registry for Load and Management of MEdicAtion OveruSE Headache (RELEASE) is an on-going nationwide, prospective, multicenter MOH registry in South Korea, which was established in January 2020 to facilitate multicenter collaborative clinical research for MOH, provide epidemiological profiles, assess the current MOH management status, and evaluate practical and effective approaches to managing MOH [24]. The RELEASE registry enrolled patients with MOH who visited 8 academic or regional headache centers participating in this registry and consented to participate. This registry regularly performs database locking and data cleaning, and this cross-sectional study was conducted on patients enrolled between April 1, 2020, and December 31, 2023. Each headache specialist of the participating centers interviewed the patients and decided whether the patients meet the study eligibility criteria. The inclusion criteria for the registry were as follows: (1) age ≥ 19 years; (2) fulfillment of MOH criteria according to the International Classification of Headache Disorders, 3rd edition [16]; (3) ability to communicate and complete questionnaires; and (4) written informed consent. Patients with any severe medical, neurological, or psychiatric conditions that impaired their ability to cooperate or understand the questionnaires were excluded.

### Ethics approval and patient consent

This study was performed in accordance with the Declaration of Helsinki and approved by the local institutional review boards of all participating centers (Approval no. of the main center: Dongtan 2020-02-004). All patients provided written informed consent to participate in the study.

### Data collection

This study used the database of the RELEASE registry, which comprised information on demographics (age, sex, body mass index, and educational level), smoking status, alcohol consumption, amount of caffeine intake per day, and medical comorbidities such as hypertension, diabetes mellitus, hyperlipidemia, heart disease, kidney disease, hepatic disease, gastric ulcer disease or reflux esophagitis, depression, fibromyalgia, and herniated disc in cervical or lumbar spine. At initial visit, each physician obtained clinical information on headache (types of primary headache disorder and chronic daily headache, headache onset, age of conversion to chronic state, age of start to overuse acute medication) and acute and/or preventive medications that patients have used. To establish the treatment strategy, each physician at participating centers interviewed the patients thoroughly with enough time. Information on acute and preventive medications was collected at the initial visit and at the one- and three-month follow-up visits. Physicians also collected monthly headache days, monthly severe headache days, and acute medication intake days, educated how to use headache diaries, and collected headache profiles at each regular (1, 3, 6, and 12 months) follow-up visit. At initial and follow-up visits, we also used a set of 9 structured questionnaires to obtain detailed data on clinical features including depression, anxiety, stress, allodynia, efficacy of acute treatment, and quality of life for all patients. Questionnaires were designed to be simple and easy to understand, regardless of educational background, ensuring the reliability of the measures. If the patient did not fully understand questions, the research coordinator of each center explained the meaning of each sentence to help them understand.

In the randomized controlled trial study [25], cured MOH was defined as no longer meeting the MOH diagnostic criteria according to the International Classification of Headache Disorders, 3rd edition [16]. However, MOH recovery in our study was defined when the patient takes less than 10 or 15 acute medications in the past month at 3 months [26] since we capture the acute medication intake days only in the past 1 month.

### Questionnaires

All patients completed a structured questionnaire designed to evaluate headache-related variables in the RELEASE study group. We used the questionnaires or scales that have validity and reliability in Korean population except the 12-item Allodynia Symptom Checklist (ASC-12). While the ASC-12 has not undergone formal validation, we utilized a Korean version of the ASC-12, which was developed through translation and back-translation, and expert review to unsure linguistic and cultural appropriateness [27]. The Headache Impact Test-6 (HIT-6) was used to assess the impact of headaches on patient’s lives. Severe impact of headache was defined as HIT-6 score ≥ 60 [28]. We also assessed headache-related disability using the Migraine Disability Assessment Scale (MIDAS) score [29]. Depression and anxiety were assessed using the Patient Health Questionnaire-9 (PHQ-9) [30] and Generalized Anxiety Disorder-7 (GAD-7) [31], respectively. The PHQ-9 includes nine questions to assess the frequency of depressive symptoms over the past two weeks. A total score of 10 or higher indicates severe depressed mood. The GAD-7, a seven-item questionnaire designed to diagnose generalized anxiety disorder, is used to assess the frequency of anxious symptoms over the past two weeks. A total score of 10 or more indicates the presence of anxiety symptoms [32, 33]. To assess the severity of allodynia, an important symptom of migraine and a factor in its chronicity, we used the 12-item Allodynia Symptom Checklist (ASC-12) [34, 35]. The ASC-12 consists of 12 questions that identify cutaneous allodynia and categorize it according to severity [35]. To assess stress, we evaluated the Perceived Stress Scale-4 (PSS-4), a self-reported questionnaire designed to measure “the degree to which individuals occur stressful situations in their lives” [36]. Finally, to evaluate the impact of headaches on quality of life, we used the Migraine Specific Quality of Life Questionnaire (MSQ), a migraine-specific instrument widely used in health-related quality of life study [37].

### Neck pain: severity, duration and timing

The presence of neck pain was assessed using a self-administered questionnaire at each visit. All patients were asked if they had neck pain, with options of ‘yes’ or ‘no’. If a patient answered ‘yes’, they were grouped as having neck pain and asked several follow-up questions about the characteristics of their neck pain. The severity of neck pain was classified as low disability/low intensity (grade 1), low disability/high intensity (grade 2), high disability/moderately limiting (grade 3), and severely limiting (grade 4), based on criteria established by the Bone and Joint Decade 2000–2010 Task Force on Neck Pain and its Associated Disorders [38, 39]. According to these criteria, severe neck pain was defined as neck pain associated with a high level of disability (grade 3 or higher) and moderate limitations in activities of daily living [40–42]. The duration of neck pain was categorized as transitory (less than a week), short duration (one week or longer but not persistent), and long duration (persistent). The temporal relationship of neck pain during different phases of a migraine attack, such as the prodrome, headache, or postdrome phase, was also assessed.

### Statistical analysis

Categorical data are presented as frequencies and percentages (%). The remaining continuous data are presented as the mean and standard deviation. For group comparisons between those with and without neck pain, the Student *t* test was used for continuous variables, while the chi-square test was used for categorical variables. No corrections for multiple testing were applied. Data analysis was performed using SPSS version 24 (SPSS, Chicago, IL, USA), and *p* < 0.05 indicated statistical significance.

## Results

### Baseline characteristics

A total of 635 patients with MOH (female, 84.1%; mean age, 46.2 ± 13.1 years) were enrolled between April 2020 to December 2023. The most common type of chronic daily headache was chronic migraine (*n* = 621, 97.8%), followed by chronic tension-type headache (*n* = 10, 1.6%) and new daily persistent headache (*n* = 3, 0.5%). Among these patients, 366 (57.6%) experienced neck pain, while 269 (42.4%) did not. In the past 3 months, 138 (37.7%) patients with neck pain experienced neck pain persistently, while 115 (31.4%) and 113 (30.9%) patients reported transitory and short duration, respectively. The majority of patients (74.9%, *n* = 274) experienced neck pain during their headache phase, while 64% (*n* = 234) and 43% (*n* = 157) of patients had neck pain before and after their headache phase, respectively. There were no significant differences in age, sex distribution, or body mass index between the two groups (Table 1). However, patients with neck pain had an earlier onset of their primary headache disorder (23.4 ± 12.7 years) compared to those without neck pain (26.2 ± 13.3 years), which was statistically significant (*p* = 0.007) (Table 1). The onset age of chronic daily headache was also significantly earlier in the neck pain group (37.9 ± 12.9 years) compared to the non-neck pain group (40.1 ± 12.8 years) (*p* = 0.037). There was no significant difference in the onset age of MOH between the two groups.

**Table 1:** Comparison of demographic and clinical characteristic between MOH patients with and without Neck Pain

	With neck pain (*N* = 366)	Without neck pain (*N* = 269)	*p*-value
Age, years	45.6 ± 12.8	47.0 ± 13.5	0.184
Female sex, n (%)	311 (85.0)	223 (82.9)	0.480
BMI, kg/m^2^	23.2 ± 4.0	23.3 ± 3.7	0.556
Onset age of primary headache, years	23.4 ± 12.7	26.2 ± 13.3	0.007
Onset age of CDH, years	37.9 ± 12.9	40.1 ± 12.8	0.037
Onset age of MOH, years	40.3 ± 12.5	41.8 ± 12.7	0.158
Monthly headache days, days	25.0 ± 5.4	24.2 ± 5.5	0.060
HIT-6	66.1 ± 7.2	65.2 ± 7.8	0.112
MIDAS	68.6 ± 63.9	62.2 ± 62.7	0.207
Depression scale (PHQ-9)	11.2 ± 6.5	10.2 ± 6.3	0.064
Anxiety scale (GAD-7)	7.4 ± 5.8	6.4 ± 5.4	0.017
Cutaneous allodynia (ASC-12)	2.4 ± 3.3	1.8 ± 3.0	0.038
Stress scale (PSS-4)	8.0 ± 2.9	7.8 ± 2.8	0.549
Quality of life scale (MSQ)	171.7 ± 70.4	184.0 ± 68.9	0.029

MOH, Medication overuse headache; BMI, Body mass index; CDH, Chronic daily headache; HIT-6, Headache impact test-6; MIDAS, Migraine disability assessment; PHQ-9, Patient health questionnaire-9; GAD-7, General anxiety disorder-7; ASC-12, Allodynia symptom checklist-12; PSS-4, Perceived stress scale-4; MSQ, Migraine-specific quality of life questionnaire

### Headache burden and associated symptoms

The monthly headache days, MIDAS score and HIT-6 score were comparable between patients with neck pain and those without neck pain. However, the neck pain group exhibited significantly higher levels of anxiety, as measured by the GAD-7 scale (7.4 ± 5.8 vs. 6.4 ± 5.4, *p* = 0.017), and more severe cutaneous allodynia, assessed by the ASC-12 scale (2.4 ± 3.3 vs. 1.8 ± 3.0, *p* = 0.038), compared to the non-neck pain group. Furthermore, patients with neck pain reported significantly poorer quality of life, as evidenced by lower scores on the MSQ (171.7 ± 70.4 vs. 184.0 ± 68.9, *p* = 0.029), suggesting a substantial impact of neck pain on their overall well-being and functioning.

### Treatment

Preventive treatment regimens showed minimal variation across initial, 1-month, and 3-month visits (Table 2). Antiepileptic drugs were the most commonly prescribed oral preventive treatment (69% at initial visit), with a significantly higher usage rate in patients with neck pain (73.5%) compared to those without (62.8%) (*p* = 0.005). Usage frequencies of other medication classes did not differ significantly based on the presence or absence of neck pain. Similar patterns were observed at the 1-month follow-up. Onabotulinum toxin administration in patients with neck pain decreased from 18.0% at the initial visit to 13.7% at the 3-month follow-up. CGRP monoclonal antibody usage showed a slight decrease in patients with neck pain (from 28.7 to 25.9%), while increasing in patients without neck pain (from 28.7 to 34.9%) over the same period.

**Table 2:** Preventive treatment regimens during the study period

	At baseline (*n* = 635)	*N* = 366	*N* = 269	At 1-month visit (*n* = 522)	*N* = 304	*N* = 218	At 3-month visit (*n* = 456)	*N* = 270	*N* = 186
	All	Neck pain (+) at baseline	Neck pain (-) at baseline	All	Neck pain (+) at baseline	Neck pain (-) at baseline	All	Neck pain (+) at baseline	Neck pain (-) at baseline
Antiepileptic drug(s), n (%)	438 (69.0)	**269 (73.5)** ^**a**^	**169 (62.8)** ^**a**^	367 (70.3)	**228 (75.0)** ^**b**^	**139 (63.8)** ^**b**^	292 (64.0)	181 (67.0)	111 (59.7)
Beta-blocker, n (%)	142 (22.4)	74 (20.2)	68 (25.3)	135 (25.9)	75 (24.7)	60 (27.5)	113 (24.8)	60 (22.2)	53 (28.5)
Calcium channel blocker, n (%)	115 (18.1)	60 (16.4)	55 (20.4)	97 (18.6)	58 (19.1)	39 (17.9)	76 (16.7)	52 (19.3)	24 (12.9)
Tricyclic antidepressant, n (%)	261 (41.1)	156 (42.6)	105 (39.0)	204 (39.1)	114 (37.5)	90 (41.3)	149 (32.7)	87 (32.2)	62 (33.3)
Onabotulinum toxinA, n (%)	123 (19.4)	66 (18.0)	57 (21.2)	94 (18.0)	49 (16.1)	45 (20.6)	75 (16.4)	37 (13.7)	38 (20.4)
CGRP mAb, n (%)	179 (28.2)	105 (28.7)	74 (27.5)	148 (28.4)	79 (26.0)	69 (31.7)	135 (29.6)	**70 (25.9)** ^**c**^	**65 (34.9)** ^**c**^
ARB/ACEi, n (%)	9 (1.4)	5 (1.4)	4 (1.5)	12 (2.3)	7 (2.3)	5 (2.3)	15 (3.3)	9 (3.3)	6 (3.2)
SNRI, n (%)	13 (2.0)	11 (3.0)	2 (0.7)	11 (2.1)	8 (2.6)	3 (1.4)	11 (2.4)	8 (3.0)	3 (1.6)
GONB, n (%)	28 (4.4)	17 (4.6)	11 (4.1)	23 (4.4)	15 (4.9)	8 (3.7)	19 (4.2)	13 (4.8)	6 (3.2)
TENS, n (%)	3 (0.5)	1 (0.3)	2 (0.7)	0	-	-	1 (0.2)	1 (0.4)	0 (0.0)
Steroid, n (%)	129 (20.3)	79 (21.6)	50 (18.6)	6 (1.1)	4 (1.3)	2 (0.9)	4 (0.9)	3 (1.1)	1 (0.5)

CGRP mAb, Calcitonin gene-related peptide monoclonal antibodies; ARB, angiotensin receptor blockers; ACEi, Angiotensin-converting enzyme inhibitors; SNRI, Serotonin norepinephrine reuptake inhibitors; GONB, Greater occipital nerve block; TENS, transcutaneous electrical nerve stimulation

^a^p-value = 0.005

^b^p-value = 0.007

^c^p-value = 0.049

### Neck pain at 3-month follow-up

**Fig. 1:**
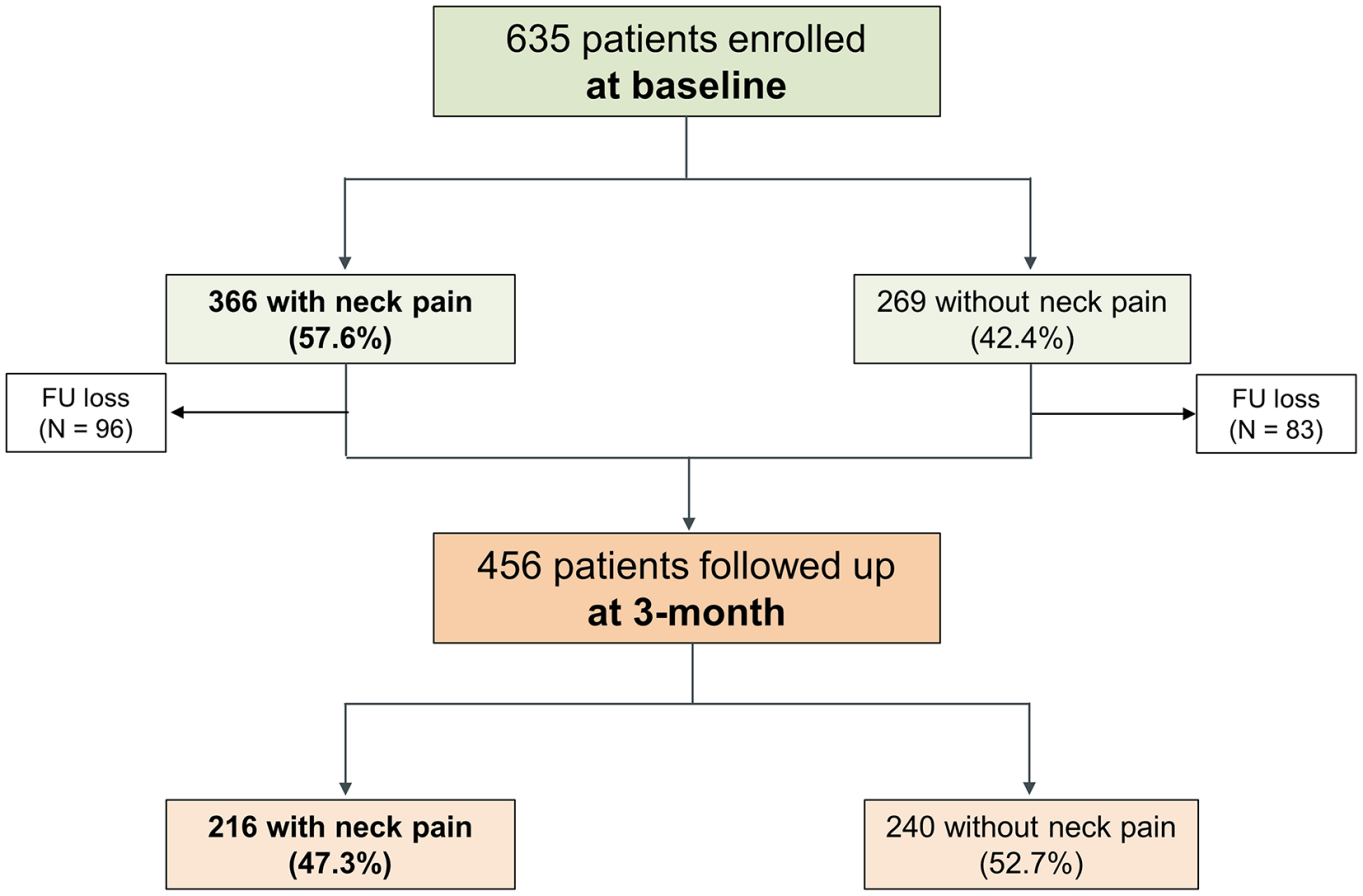
Flow Chart of Neck Pain Prevalence in MOH patients at Baseline and 3-Month Follow-up. RELEASE, The Registry for Load and Management of MEdicAtion OveruSE Headache; MOH, Medication overuse headache; FU, Follow-up

At the 3-month follow-up, 456 patients returned, resulting in a follow-up rate of 71.8%. Among the 270 patients who initially reported neck pain, 195 (72.2%) continued to experience neck pain, while 75 (27.8%) no longer reported neck pain. Conversely, among the 186 patients without baseline neck pain, 21 (11.3%) developed neck pain of varying severity levels (Fig. 1). In the 456 patients who completed the follow-up, there was a decrease in the overall presence of neck pain from 57.6% at baseline to 47.3% at 3 months (*p* = 0.157). Notably, the rate of severe neck pain (defined as grade 3 or greater) decreased from 40.4% at baseline to 19.4% at 3 months (*p* < 0.001). A total of 257 patients (56.4%) had recovered from MOH. Among the patients followed up at 3 months, a lower proportion of those with baseline neck pain recovered from MOH compared to those without neck pain (47.8% vs. 68.8%, *p* < 0.001). The proportion of patients experiencing severe neck pain was significantly lower among those who recovered from MOH (4.7%) compared to those who had not recovered (15.1%) (*p* < 0.001) (Table 3).

**Table 3:** Comparison of clinical characteristic with and without MOH Recovery patients at 3-Month follow-up

	With MOH recovery (*N* = 257)	Without MOH recovery (*N* = 199)	*p*-value
Age, years	46.4 ± 13.1	46.7 ± 12.9	0.821
Female sex, n (%)	218 (84.8)	174 (87.4)	0.497
BMI, kg/m^2^	23.5 ± 4.1	23.0 ± 3.7	0.154
Severe Neck pain (%)	12 (4.7%)	30 (15.1%)	0.001
Persistent of Neck pain (%)	21 (8.2%)	21 (10.6)	0.801
HIT-6	65.4 ± 7.4	65.9 ± 7.4	0.499
MIDAS	67.4 ± 62.5	72.7 ± 71.2	0.405
Depression scale (PHQ-9)	9.9 ± 6.1	11.7 ± 6.8	0.003
Anxiety scale (GAD-7)	6.3 ± 5.2	7.8 ± 5.9	0.005
Cutaneous allodynia (ASC-12)	2.0 ± 3.3	2.3 ± 3.0	0.401
Stress scale (PSS-4)	7.7 ± 2.8	8.2 ± 3.0	0.085
Quality of life scale (MSQ)	183.5 ± 70.4	169.3 ± 70.4	0.032

MOH, Medication overuse headache; BMI, Body mass index; HIT-6, Headache impact test-6; MIDAS, Migraine disability assessment; PHQ-9, Patient health questionnaire-9; GAD-7, General anxiety disorder-7; ASC-12, Allodynia symptom checklist-12; PSS-4, Perceived stress scale-4; MSQ, Migraine-specific quality of life questionnaire

**Fig. 2:**
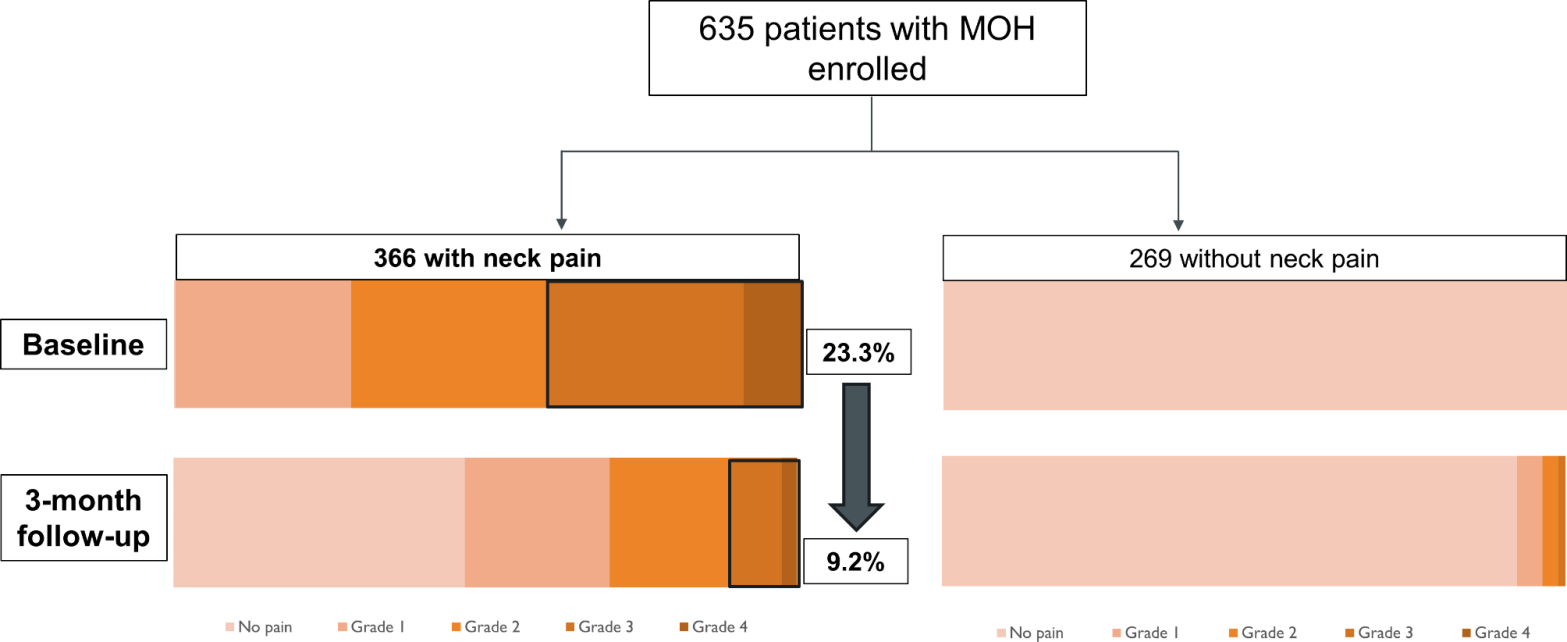
Horizontal Bar Graph of Severity in MOH patients at Baseline and 3-Month Follow-up: In the neck pain group, the percentage of patients with grade 3 or greater severity decreased to 9.2% at 3-month follow-up compared to 23.3% at baseline. MOH, Medication overuse headache

## Discussion

This prospective, nationwide, multicenter study investigated the connection between neck pain and MOH and reported several key findings. The incidence of neck pain is quite high and associated with a substantial burden (earlier onset of the primary headache disorder, greater headache-related disability, higher level of anxiety, and cutaneous allodynia. Treating MOH reduced the proportion of severe neck pain (grade 3 and 4), especially in patients who recovered from MOH. This study investigated the connection between neck pain and MOH, a disabling secondary headache disorder that has received relatively limited research attention. Our findings reveal that 57.6% of the MOH patients reported concurrent neck pain at baseline. This observation aligns with previous studies documenting a strong comorbidity between neck pain and primary headache disorders such as migraines and tension-type headaches [1, 43], suggesting that this association may extend to secondary headache conditions like MOH.

The prevalence of neck pain in the general population has been reported to be 2,696.5 per 100,000 [44]. However, among chronic migraine patients, the prevalence of neck pain is substantially elevated, reaching 87% [45]. In our study, the prevalence of neck pain among patients with chronic migraine was 58.0%, which is much lower than that in the previous study, even considering the wide range of neck pain prevalence (65–100%) reported in previous studies [4, 46–49]. This discrepancy in prevalence may be attributed to (1) potential patient misattribution of neck pain as unrelated to their headaches, resulting in under-reporting, and (2) possible insufficiency in interviewer-patient communication, leading to incomplete capture of symptom.

Regarding the timing of neck pain, previous literature has reported that 31.6% [9] and 85% [13] of migraine patients experience neck pain in the premonitory phase, respectively. In addition, in some studies, 32% and 57% of patients reported neck pain as a migraine trigger factor [13, 50]. Given that there is no clear distinction between migraine triggers and premonitory symptom [9], the neck pain in these previous studies could have been a premonitory symptom of migraine. In our study, 64%, 75%, and 43% of patients with neck pain reported neck pain in the premonitory, headache, and post-dromal phases, respectively, which is not significantly different from previous studies.

Notably, MOH patients with neck pain in this study exhibited an earlier onset of their primary headache disorder (23.4 ± 12.7 years) and chronic daily headache (37.9 ± 12.9 years) compared to those without neck pain (onset age of primary headache disorder: 26.2 ± 13.3 years, onset age of chronic daily headache: 40.1 ± 12.8 years). This finding suggests that the presence of neck pain may be associated with a more severe and prolonged clinical course, potentially contributing to the development of medication overuse and the subsequent transition to MOH. However, the exact underlying mechanisms linking neck pain to an earlier headache onset and chronification of headache require further investigation. It is important to note that these factors may not be directly related, and alternative explanations for their co-existence should be considered. Previous studies reported that upper cervical spine dysfunction, including the presence of myofascial trigger points, reduced neck mobility, and head forward posture in migraine patients, may be a factor in initiating and maintaining migraine attacks [51–53]. In detail, Gerwin [54] and Simons et al. [55] suggested that pain in the muscles of the pericranial head, neck, and shoulders may be referred to the head and manifest as a headache. However, this mechanism should be distinguished from the actual progression of migraine attacks. Migraine attacks may initially manifest as neck pain before evolving into a typical migraine headache. Hu et al. [56] reported that noxious stimulation of muscle afferents increases the excitability of spinal cord neurons, and Boquet et al. [57] found that in 24 subjects with strictly unilateral migraine, upper cervical spine dysfunction was located ipsilateral to the migraine. The trigeminocervical complex (TCC) plays a crucial role in the mechanism of neck pain in chronic migraine. The TCC functions as a unit within the upper cervical spinal cord, where sensory nerve fibers from the trigeminal nerve converge with input from the upper cervical nerves [58]. This convergence allows pain signals to be transmitted bidirectionally between the head and neck. Sensitization of the TCC can be triggered by central and peripheral sensitization [59–61], neuroinflammatory changes [62], and muscular factors [63], resulting in pain. This mechanism likely contributes to musculoskeletal factors involved in neck pain associated with chronic migraine.

In our study, MOH patients with neck pain demonstrated higher level of headache-related disability, anxiety and cutaneous allodynia, as well as lower quality of life compared to those without neck pain. These findings are consistent with previous studies on migraine and tension-type headache patients, which had shown associations between neck pain and increased headache-related disability, anxiety, depression, and reduced quality of life [11, 64–66]. These observations emphasize the detrimental impact of neck pain on various aspects of daily functioning and underscore the need for increased attention to and management of neck pain, which has often been overlooked in headache treatment.

At 3-month follow-up, the proportion of severe neck pain was significantly (40.4% vs. 19.4%, *p* < 0.001) compared to baseline, while the presence of neck pain showed 10%-point decrease without statistical significance (57.6% vs. 47.3%, *p* = 0.157). The decrease in severity of neck pain might be due to reduced central sensitization [67], decreased tension in the neck and shoulder muscles [68], and reduced psychological factors leading to success in cease overuse of medications [69]. These findings highlight the significance of neck pain in MOH patients. While assessing neck pain may be important, the optimal management approach remains unclear and requires further investigations and consideration in long-term management strategies.

Twenty-one patients reported neck pain at 3 months who did not report neck pain at initial visit. After the initial visit, patients used headache diaries to collect the headache profiles including neck pain, which can allow us to obtain more accurate information. At initial visit, however, patients should recall whether they had experienced neck pain, and those with minimal or mild neck pain might not recall that they had neck pain even though they actually had. Among 21 patients who reported neck pain at 3 months but did not at initial visit, only 3 patients had grade 3 neck pain and no patient reported grade 4 neck pain at 3 months.

Our study has certain limitations. First, it used cross-sectional data at baseline, which limits the ability to establish causal relationships between neck pain and MOH. A longitudinal design following patients over time would provide stronger evidence for the temporal relationship and potential bidirectional influences. Second, the assessment of neck pain, its severity, and other clinical characteristics relied on self-reported questionnaires and measures. While validated scales were used, subjective self-reporting may introduce potential biases and inaccuracies in data collection. Third, the study focuses solely on MOH patients and does not include a control group of individuals without headache disorders or those with primary headache disorders like migraine. The inclusion of appropriate control groups would allow for more robust comparisons and help distinguish the specific effects of MOH versus other headache types on neck pain prevalence and severity. Fourth, our study lacks the assessment of musculoskeletal impairments that could explain the nature of neck pain. Understanding these impairments is crucial for elucidating the origin of neck pain and identifying possible shared mechanisms of improvement. Finally, the 3-month follow-up assessment may not be sufficient to capture the full extent of changes in neck pain severity and its potential impact on headache outcomes over a longer period.

## Conclusion

In conclusion, the presence of neck pain in MOH patients was associated with an earlier onset of their primary headache disorder, chronic daily headache, higher levels of anxiety, cutaneous allodynia, and poorer quality of life, despite no significant differences in headache frequency or disability scores. This study also demonstrated a correlation between neck pain improvement and MOH recovery, suggesting the potential importance of considering neck pain in the clinical assessment and management of MOH. Further research is needed to understand mechanism between neck pain and MOH, which could inform more detailed treatment strategies.

## Data Availability

No datasets were generated or analysed during the current study.

## Abbreviations

MOH: Medication overuse headache
RELEASE: The REgistry for Load and management of MEdicAtion overuSE headache
HIT-6: The Headache Impact Test-6
MIDAS: The Migraine Disability Assessment Scale
PHQ-9: The Patient Health Questionnaire-9
GAD-7: The Generalized Anxiety Disorder-7
ASC-12: The 12-item Allodynia Sympom Checklist
MSQ: The Migraine Specific Quality of Life Questionnaire
